# Dental pulp mesenchymal stem cell (DPSCs)-derived soluble factors, produced under hypoxic conditions, support angiogenesis via endothelial cell activation and generation of M2-like macrophages

**DOI:** 10.1186/s12929-024-01087-6

**Published:** 2024-11-04

**Authors:** Ludovica Barone, Martina Cucchiara, Maria Teresa Palano, Barbara Bassani, Matteo Gallazzi, Federica Rossi, Mario Raspanti, Piero Antonio Zecca, Gianluca De Antoni, Christina Pagiatakis, Roberto Papait, Giovanni Bernardini, Antonino Bruno, Rosalba Gornati

**Affiliations:** 1 Laboratory of Cell Biology, Department of Biotechnology and Life Sciences, University of Insubria, 21100 Varese, Italy; 2 Laboratory of Immunology and General Pathology, Department of Biotechnology and Life Sciences, University of Insubria, 21100 Varese, Italy; 3 Laboratory of Innate Immunity, Unit of Molecular Pathology, Biochemistry and Immunology, Istituto di Ricovero e Cura a Carattere Scientifico (IRCCS) MultiMedica, 20138 Milan, Italy; 4 Department of Medicine and Technological Innovation, University of Insubria, 21100 Varese, Italy

**Keywords:** Dental pulp stem cells, Mesenchymal stem cells, Angiogenesis, Secretome, Tissue engineering, Macrophage polarization, Cell-free device

## Abstract

**Background:**

Cell therapy has emerged as a revolutionary tool to repair damaged tissues by restoration of an adequate vasculature. Dental Pulp stem cells (DPSC), due to their easy biological access, ex vivo properties, and ability to support angiogenesis have been largely explored in regenerative medicine.

**Methods:**

Here, we tested the capability of Dental Pulp Stem Cell-Conditioned medium (DPSC-CM), produced in normoxic (DPSC-CM Normox) or hypoxic (DPSC-CM Hypox) conditions, to support angiogenesis via their soluble factors. CMs were characterized by a secretome protein array, then used for in vivo and in vitro experiments. In in vivo experiments, DPSC-CMs were associated to an Ultimatrix sponge and injected in nude mice. After excision, Ultimatrix were assayed by immunohistochemistry, electron microscopy and flow cytometry, to evaluate the presence of endothelial, stromal, and immune cells.

For in vitro procedures, DPSC-CMs were used on human umbilical-vein endothelial cells (HUVECs), to test their effects on cell adhesion, migration, tube formation, and on their capability to recruit human CD14^+^ monocytes.

**Results:**

We found that DPSC-CM Hypox exert stronger pro-angiogenic activities, compared with DPSC-CM Normox, by increasing the frequency of CD31^+^ endothelial cells, the number of vessels and hemoglobin content in the Ultimatrix sponges. We observed that Utimatrix sponges associated with DPSC-CM Hypox or DPSC-CM Normox shared similar capability to recruit CD45^−^ stromal cells, CD45^+^ leukocytes, F4/80^+^ macrophages, CD80^+^ M1-macrophages and CD206^+^ M2-macropages. We also observed that DPSC-CM Hypox and DPSC-CM Normox have similar capabilities to support HUVEC adhesion, migration, induction of a pro-angiogenic gene signature and the generation of capillary-like structures, together with the ability to recruit human CD14^+^ monocytes.

**Conclusions:**

Our results provide evidence that DPSCs-CM, produced under hypoxic conditions, can be proposed as a tool able to support angiogenesis via macrophage polarization, suggesting its use to overcome the issues and restrictions associated with the use of staminal cells.

**Supplementary Information:**

The online version contains supplementary material available at 10.1186/s12929-024-01087-6.

## Background

Following tissue injury, angiogenesis, defined as the formation of new blood vessels from a pre-existing vasculature, is required to re-vascularize and ensure oxygen and nutrient delivery to the damaged tissues. Also, angiogenesis supports the migration of other cells involved in the healing process, such as fibroblasts, mesenchymal cells, and immune cells, to the site of injury. These cells cooperate for the synthesis and deposition of extracellular matrix (ECM) components, contributing to tissue remodeling and restoration of morpho-structural integrity.

Non-endothelial cells, such as stromal cells and other type of cells (including immune cells), recruited to the damaged area, can generate a unique microenvironment, via soluble factors, [1–6] that remodels the surrounding matrix to support suitable cellular processes following a trauma [7, 8]. Among these, macrophages represent the typical prototype of non-endothelial cells, contributing to angiogenesis during tissue repair [9–12]. Based on their cellular plasticity, macrophages can undergo the so-termed M2-like polarization, generating immune cells able to secrete VEGF and TGFβ, two crucial cytokines to promote, respectively, endothelial and fibroblast activation [9–12].

Mesenchymal stem cells (MSCs) are a population of multipotent stem cells that possess self-renewal capabilities and the potential to differentiate into various cell lineages of mesodermal origin. MSCs can be derived from various biological sources such as bone marrow [13], adipose tissue [14], umbilical cord tissue, dental pulp [15, 16] and amniotic fluid [17], offering high accessibility, efficient isolation, and maintenance in ex vivo conditions.

Numerous studies have highlighted the significant role of angiogenesis in tissue repair and healing across various organs and tissues, including skin, bone, muscle, and organs such as heart and liver [18, 19]. Therapeutic strategies aimed at enhancing angiogenesis have shown promise in promoting tissue regeneration and improving wound healing outcomes [20, 21].

MSCs have gained significant attention in regenerative medicine and tissue engineering due to their unique characteristics and therapeutic potential, including their capability to induce angiogenesis. Accordingly, diverse MSC-based randomized clinical trials for ischemic heart disease therapy are being registered and completed [22–29]. While the therapeutic potential of MSCs in a re-vascularization approach appears as a relevant tool in the field of regenerative medicine, there are still some restrictions and limitations for their direct employment in clinical settings. Major concerns for the use of MSCs in regenerative medicine include cell mortality rate, anti-angiogenic activities [30–32], and immunosuppressive functions [33–35].

Based on these limitations, several approaches, such as the use of MSC-derived products, including their soluble factors (secretome), have been explored. The MSC secretome defines a plethora of soluble growth factors (GF) and cytokines that guide proliferation, adhesion, and differentiation of stem and progenitor cells towards the formation of functional vascular networks [36, 37]. In line with this view, we recently demonstrated that adipose-derived MSCs (ASCs), as a whole cell preparation, cell extracts or by using their secretome, once associated with a scaffold, exhibit similar pro-angiogenic activities in vivo [38]. Also, we found that ASC-CMs generated in hypoxic conditions have a secretome enriched in pro-angiogenic soluble factor and exhibit, both in vivo and in vitro, stronger pro-angiogenic activities compared to ASC-CM obtained in normoxic conditions [38].

Dental pulp stem cells (DPSCs) identify a unique cell population embedded within the pulp cavity of the impacted third molars. DPSCs were firstly isolated and characterized by Gronthos et al. [15] and subsequently explored for their potential use in regenerative medicine.

Here, we investigated the pro-angiogenic activities of soluble secreted factors, namely conditioned media (CMs) of DPSCs, generated under normoxic and hypoxic conditions, in supporting in vitro and in vivo angiogenesis.

We found that CMs from DPSCs in hypoxic conditions exhibit higher pro-angiogenic activities, in vivo, by recruiting endothelial cells and M2-like macrophages and generating a mature morpho-functional vascular network.

Also, CMs from DPSCs in hypoxic conditions were able to activate a pro-angiogenic transcriptome, increase cell migration, adhesion, and formation of capillary-like structures by human umbilical-vein endothelial cells (HUVECs), compared to CMs of DPSCs in normoxic condition.

Finally, CMs from DPSCs in hypoxic conditions increased monocyte recruitment and M2-like macrophage polarization, in vitro, compared to CMs of DPSCs in normoxic condition.

## Materials and methods

### Ethics approval and consent to participate

Dental pulp-derived mesenchymal stem cells (DPSCs) were isolated from dental pulp tissues of 3 healthy subjects (two males and a female), undergoing third molar extraction. The subjects provided their informed consent, to be included in the study and were naïve for treatments at the time of the medical procedure. The study was approved by the institutional review board ethics committees. Subjects were recruited within a clinical protocol by “Ospedale di Circolo Fondazione Macchi” and approved by the institutional Ethical Committee (protocol n° 0034086, 9-10-2013) according to the Helsinki Declaration of 1975, as revised in 2013.

CD14^+^ monocytes were isolated from blood samples of healthy-donor volunteers recruited within the protocol n° 463.2021, approved by the IRCCS MultiMedica internal Ethical Committee, according to the Helsinki Declaration of 1975 as revised in 2013.

In vivo studies used male athymic BALB/c nude Crl:CD1-Foxn1^nu086^ mice (Charles River mice, seven weeks); Mice were housed under standard conditions with a 12-h light/dark cycle and provided ad libitum access to food and water. The experiments were conducted in compliance with the guidelines established by the Italian and European Community (D.L. 2711/92 No.116; 86/609/EEC Directive), adhering to the principles of the 3 Rs (Replacement, Reduction, and Refinement) and carried out within an approved protocol by the institutional ethics committee.

### Generation of conditioned media (CM)

**Fig. 1:**
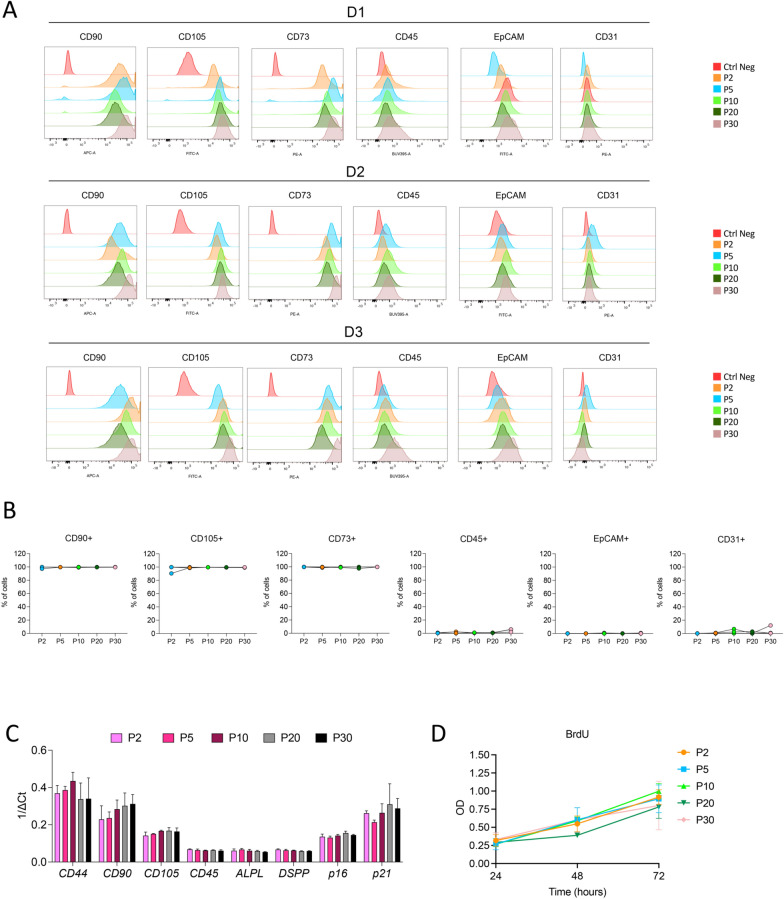
Characterization of DPSCs. DPSCs at different culture passages (P2-P30) were characterized by (**A, B**) flow cytometry for CD90, CD105, CD73, CD45, EpCAM, CD31 surface markers; (**C**) real-time PCR for CD44, CD90, CD105, CD45, ALPL, DSPP, p16, p21 gene expression; (**D**) BrdU assay, for their proliferation rate

DPSC-CMs were prepared following the previously described method, as in [39]. In brief, when the cells at 5th passage reached 70–80% confluence, media were removed, and cells were washed twice with PBS. Cells were incubated in fetal bovine serum (FBS)-free Dulbecco’s Modified Eagle Medium (DMEM), for 72 h in normoxic (21% O_2_) or hypoxic (2% O_2_) conditions. The parameters used for hypoxia condition were: 2% O_2_, 5% CO_2_, 93% N_2_. No color changes were observed in culture medium, during the 72 h of starvation, both in normoxic and hypoxic conditions, that together with the proliferation rate (showed in Fig. 1) and cell morphology (Supplementary Fig. 1), confirm a healthy state of DPSCs. The media were then removed and centrifuged at 1000xg for 10 min, to deplete eventual cell debris. To maximize the protein content, collected CMs were concentrated, using the Amicon Ultra 15 mL Centrifugal filter device (Millipore, Darmstadt, Germany) with a 3 kDa cut-off, according to the manufacturer’s instructions. 13 mL of DPSC-CMs were loaded into the tubes and centrifuged at 5000×g for 60 min at 4 °C. The concentrated media were collected, quantified in term of total protein content, and then stored at −80 °C until use for in vivo and in vitro experiments.

### Flow cytometry

DPSCs isolated from three donors (see supplementary methods), were seeded in T25 flasks, collected at passages 2, 5, 10, 20, and 30 and characterized by flow cytometry. Briefly, DPSCs were stained for 30 min 4 °C, with the following anti-human monoclonal antibodies (all purchased from BD Biosciences): APC-CD90 (BD clone 5E10), BUV395-CD45 (BD clone HI30), FITC-EpCAM (BD clone EBA-1), PE-CD31 (BD clone WM59), PE-CD73 (BD clone AD2). Viable cells were identified based on doublet exclusion (side scatter area/SSC-A Vs side scatter height/SSC-H) and their morphology (forward scatter area/FSC-A Vs side scatter area/ SSC-A), then interrogated for the fluorescence signals associated to the selected surface antigens.

Following excision from mice, Ultimatrix sponges were filtered, using a 70 μm pore cell strainer (BD Biosciences), using a syringe plugger until complete plug dispersion, to obtain a single cell suspension for flow cytometry analysis. The single cell suspension was stained for 30 min, 4 °C, with the following anti-mouse monoclonal antibodies (all purchased from BD Biosciences): FITC-CD31 (clone: MEC 13.3), BUV-395-CD45 (clone: 30-F11), PE-CF594-F4/80 (clone: T45-2342), BV-421-CD80 (clone: 16-10A1), Alexa Fluor-647-CD206 (clone: MDR5D3). Samples were acquired using a FACS Fortessa × 20 (BD Biosciences), equipped with 5 lasers. Viable cells were identified based on doublet exclusion (side scatter area/SSC-A Vs side scatter height/SSC-H) and their morphology (forward scatter area/FSC-A Vs side scatter area/ SSC-A). Viable cells were used to identify different cell types as follows: CD45^−^ cells (stromal cells), CD45^−^CD31^+^ cells (endothelial cells), CD45^+^ cells (total leukocytes), CD45^+^F4/80^+^ cells (total macrophages), CD45^+^F4/80^+^CD80^+^ cells (M1-like macrophages), CD45^+^F4/80^+^CD206^+^ (M2-like macrophages). FACS data were exported as FCS files and analyzed with the FlowJo v10 software (BD Biosciences).

### RNA extraction, reverse transcription, and real-time PCR

DPSCs from the three donors were collected at passages 2, 5, 10, 20, and 30 and characterized by qPCR for stem markers *CD44, CD90*, and *CD105*, leukocyte marker *CD45*, differentiation markers Alkaline Phosphatase (*ALPL*) and Dentin Sialophosphoprotein (*DSPP*), cell senescence markers* p16* and *p21*. Procedures for RNA extraction, RT and qPCR are detailed in supplementary methods.

HUVECs, following 6 h of stimulation with DPSC-CMs, were collected in trizol reagent, and stored at − 80 °C until further use. The *18S* RNA gene was used as a housekeeping gene, and HUVECs or macrophages, cultured in serum-free media, served as the baseline control. Procedures for RNA extraction, RT and qPCR are detailed in supplementary methods.

### BrdU assay

To assess the effect of long-term passaging on DPSCs, cell proliferation was evaluated using the kit BrdU cell proliferation ELISA (Roche Life Sciences, Switzerland). DPSCs from the three donors, collected at passages 2, 5, 10, 20, and 30, were used. The experiment was conducted following the manufacturer’s instructions: briefly, 200 cells/well were seeded onto a 96-well plate and incubated for 24 h at 37 °C and 5% CO_2_. Once cells adhered to the plate, BrdU labelling solution was added and incubated for 24 h. Afterwards, medium was removed and cells were fixed with FixDenat solution for 30 min at RT; anti-BrdU-POD working solution was then added and incubated for 90 min at RT. Wells were then washed three times with washing solution before adding the Substrate solution for 15 min, until color development was sufficient for detection. Afterwards, 1 M H_2_SO_4_ was added to each well and the absorbance was measured at 450 nm using the GloMax^®^ Discover Microplate Reader (Promega, Milano, Italy). The same process was repeated for 24 h, 48 h, and 72 h.

### In vivo Ultimatrix sponge assay

DPSC-CMs were prepared following the previously described method, as reported in [39].The effects of DPSC-CMs, obtained in normoxia or hypoxia, on angiogenesis in vivo, was tested using the Ultimatrix sponge assay, as in [38]. The procedure for Ultimatrix sponge preparation has been detailed in Supplementary Methods section.

### Characterization of DPSC-conditioned media

The DPSC-CMs were characterized, in term of soluble factor content, using the Human Cytokine Array C7 (RayBiotec) (Peachtree Corners, GA, USA), starting from 50 μg of total protein of DPSC-CMs from normoxic or hypoxic conditions and following the manufacturer’s indication and as in [38, 40, 41].

### Hemoglobin quantification

Hemoglobin content in excised sponges was determined using the colorimetric Drabkin’s assay. Briefly, sponges were mechanically processed in 300 μL of 1 × PBS (Euroclone). 200 μL of the supernatant were collected ad added to 800 μL of Drabkin’s solution and incubated at room temperature, protected from light, for 20 min. 100 μL of the incubated solution was transferred into a 96 well plate and read at 595 nm, using a SpectraMax plate reader.

### Optical microscopy

The formation of new vessels inside the scaffolds was observed by optical microscopy as described in [42]. Samples, fixed in 4% PFA at RT for 2 h, were embedded in paraffin, following sequential dehydration with ethanol (70, 80, 90, 95, 100%), and cut using an RMC-RM3 rotary microtome (TiEsseLab, Milan, Italy). Slides were then stained with hematoxylin and eosin (H&E) solutions, following classical procedures, and finally analyzed. Vessels were counted from five nonconsecutive section (5 µm), considering three-microscope fields per section, using ISCapture software (version 3.6.9.4).

### Scanning electron microscopy (SEM) analysis

Samples embedded in paraffin were cut (15–30 μm slices) using an RMC-RM3 rotary microtome (TiEsseLab, Milan, Italy) and placed on slides for SEM observation.

Specimens were dehydrated using a series of ethanol concentrations and dried using hexamethyldisilazane (Sigma Aldrich, Milano, Italy). Subsequently, they were coated with a 10 nm layer of gold using the Emitech K550 system and examined using a Philips SEM-FEG XL-30 electron microscope (Eindhoven, The Netherlands).

### Stimulation of endothelial cells with CMs

3 × 10^5^ HUVECs (see supplementary methods for culturing and maintenance) seeded in six well plates, were exposed to 50 μg of total protein from DPSC-CMs, obtained under normoxic and hypoxic conditions, in serum-free media, for 6 h.

### Cell migration

The capability of DPSC-CMs (Normox or Hypox) to influence the migration of HUVEC or CD14^+^ monocytes (see supplementary methods for human CD14^+^ monocyte isolation) were tested by transwell assay.

For endothelial cell migration, 2 × 10^4^ HUVECs were placed on the upper chamber of 24-well transwell (Corning), with a 10 μm pore filter cut-off coated with 2 μg of human fibronectin (Sigma Aldrich). 50 μg/well of DPSC-CMs, or 1:4 diluted (Normox or Hypox), or the single cytokines IL-6 (50 ng/mL), IL-8 (20 ng/mL), SDF-1 (100 ng/mL), or the IL-6 + IL-8 + SDF-1 combination, were used to induce endothelial cell migration. EBM starvation of complete medium was used as negative and positive internal controls.

For monocyte migration, 25 × 10^4^ CD14^+^ monocytes were placed on the upper chamber of 24-well transwell (Corning), with a 5 μm pore filter cut-off, respectively, coated with 2 μg of human fibronectin (Sigma Aldrich). 50 μg/well of DPSC-CMs (Normox or Hypox) were used to induce cell migration. RPMI starvation of complete medium was used as negative and positive internal controls.

Transwells were incubated at 37 °C, 5% CO_2_ for 6 h. Upper chambers were collected, washed in PBS, fixed with 4% PFA for 10 min, at RT, then washed, stained with 10 μg/mL Hoechst 33342 for 15 min, washed in PBS, and finally acquired using a fluorescence microscope (Leica). The number of fluorescent cells, as readout of migration, were counted using the ImageJ software. Tree blind fields for each filter were acquired and summed to estimate the number of migrated cell/filter/conditions.

### Tube formation

The ability of DPSC-CMs (Normox or Hypox) to induce a capillary-like network in vitro, was tested by tube formation assay on HUVECs. HUVECs (8 × 10^4^ cells/well) were seeded in a 24-well plate, previously coated with 50 µL of 10 mg/mL polymerized phenol red-free, reduced growth factors Matrigel (BD). Following exposure to 50 µg/mL of DPSC-CMs Normox or DPSC-CMs Hypox, in serum-free EBM medium, HUVECs were incubated at 37 °C, 5% CO_2_ for 24 h. EBM starvation or complete medium were used as negative and positive internal controls.

The formation of capillary-like structures was detected by microphotographs, using an inverted microscope (Leica). The number of master segments, total master segment length, number of meshes and total mashes area, as readouts of tube formation efficiency, were determined, using ImageJ software and the Angiogenesis Analyzer tool.

### Statistical analysis

Results were analyzed using the GraphPad Prism software v10 (GraphPad Prism Inc., San Diego, CA, USA). Data are shown as means ± SEM, Student t-test or One-Way ANOVA, followed by Tukey’s post-hoc test correction. P values ≤ 0.05 were considered statistically significant.

## Results

### Characterization of DPSCs

DPSCs were characterized using flow cytometry (FACS), qPCR, and BrdU assays. FACS analysis revealed that isolated DPSCs maintained the expression of the MSC markers CD90, CD105, CD73 at all the passages tested (P2, P5, P10, P20, P30) and for the three donors (D1-3), with no contaminations of CD45^+^ cells (leukocytes), EpCAM^+^ cells (epithelial cells), and CD31^+^ cells (endothelial cells) (Fig. 1A, B). FACS results were corroborated by qPCR analysis, confirming the maintenance of the stable expression of *CD90* and *CD105* markers, in addition to *CD44*, while low levels of *CD45*, *ALPL*, *DSPP* were found for all the passages tested (P2, P5, P10, P20, P30) and for all three donors (D1-3) (Fig. 1C). Finally, qPCR analysis for *P16* and *P21*, genes associated with senescence, showed similar expression levels (Fig. 1C), as confirmed by the comparable proliferation capabilities (detected by BrdU assay, Fig. 1D), independently from passages and donors.

### CMs from DPSCs support angiogenesis in vivo

**Fig. 2:**
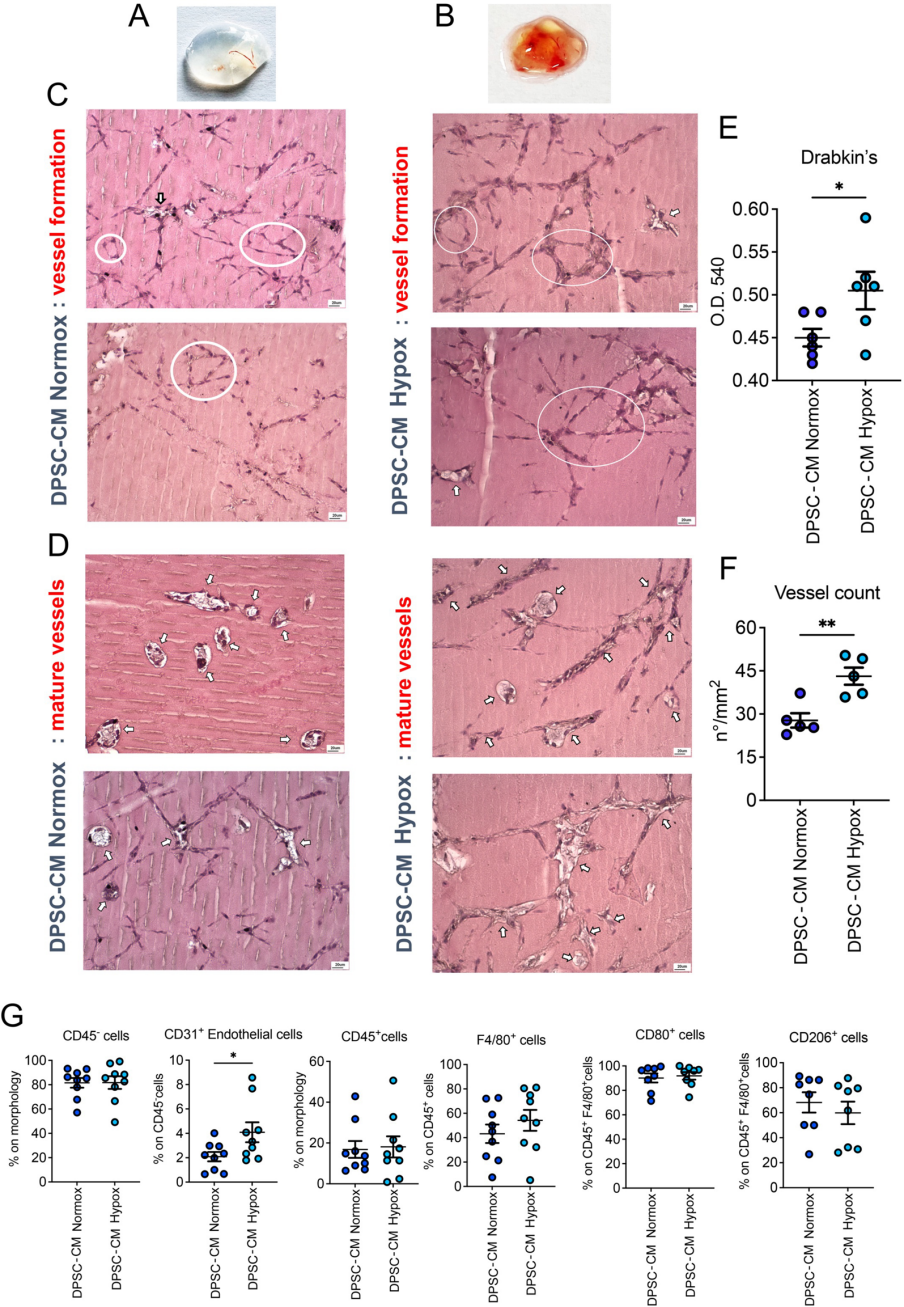
In vivo pro-angiogenic effects of DPSC-CMs produced in normoxia and hypoxia*.* The pro-angiogenic effects of DPSC-CMs were investigated by Ultimatrix sponge assay. **A**, **B** Representative excised Ultimatrix sponges associated with DPSC-CM Normox and DPSC-CM Hypox; **C**, **D** representative H&E images of sections from Ultimatrix sponges associated with DPSC-CM Normox and DPSC-CM Hypox showing vascular-like structures (circles) and mature blood vessels (arrows) respectively. Scale bar 20 μm; **E** hemoglobin content, detected by Drabkin’s assay, in sponges associated with DPSC-CM Normox and DPSC-CM Hypox; **F** vessel count in Ultimatrix sponges associated with DPSC-CM Normox and DPSC-CM Hypox; **G** flow cytometry analysis for the content of stromal CD45^−^ cells, CD31^+^ endothelial cells, CD45^+^ total leukocytes, total F4/80^+^ macrophages, CD80^+^ M1 and CD206^+^ M2 macrophages in sponges associated with DPSC-CM Normox and DPSC-CM Hypox. Results are shown as mean ± SEM, t-student test, *p ≤ 0.05; **p ≤ 0.01. Experiments were performed using CMs from 3 donors

Based on our previous results on ASCs [38], we tested, in vivo, the capabilities of DPSC-CM Normox or DPSC-CM Hypox to support angiogenesis when associated with the Ultimatrix sponge [38]. Morphological inspection of excised Ultimatrix plugs clearly showed higher vascularization with DPSC-CM Hypox, compared to DPSC-CM Normox (Fig. 2A, B): these data were confirmed by histological analysis that showed vessel formation (Fig. 2C) and mature vessels (Fig. 2D). Drabkin’s assay revealed that DPSC-CM Hypox showed statistically significant increased levels of hemoglobin (Fig. 2E) together with an increased number of blood vessels (Fig. 2F), compared to DPSC-CM Normox. FACS results confirmed the capability of DPSC-CM Hypox to increase the recruitment of CD31^+^ endothelial cells, compared to DPSC-CM Hypox (Fig. 2G), while similar capabilities to recruit CD45^−^ stromal cells, CD45^+^ leukocytes, F4/80^+^macrophages, CD80^+^M1 and CD206^+^M2 macrophages were observed for both CM formulations (Fig. 2G).

**Fig. 3:**
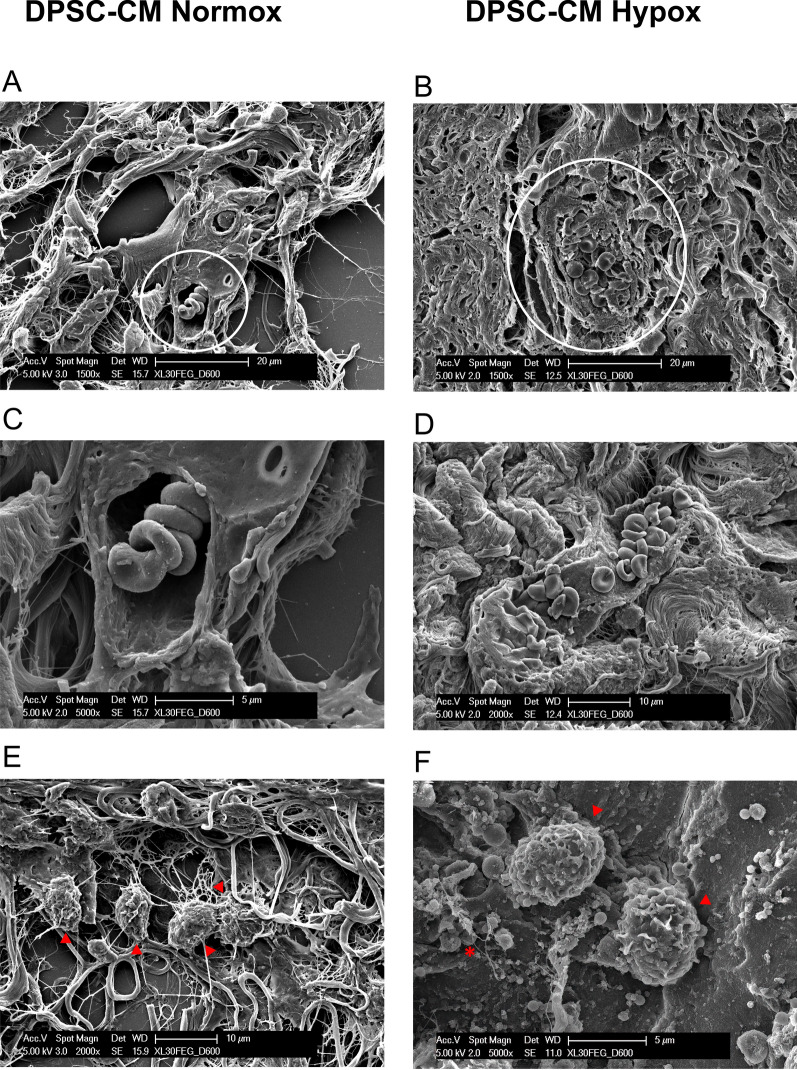
Ultrastructural analysis of DPSC-CM Ultimatrix sponges. Representative SEM images of sponges associated with DPSC-CM Normox (**A, C, E**) and DPSC-CM Hypox (**B, D, F**) are showed. Beside the collagen fibrils, is evident the presence of blood vessels, full of erythrocytes, within the scaffold (circle) (**A, B**). A magnification of the blood vessel in the picture **A** is shown in **C**. A capillary, full of erythrocytes, in longitudinal section, is represented in figure **D**. Macrophages (arrowheads) (**E, F**) and platelets (asterisk) (**F**) were also present. Scale bars are indicated in the pictures

Moreover, ultrastructural analysis corroborates these findings (Fig. 3A–F). SEM images of sponges associated with DPSC-CM Normox (A, C, E) and DPSC-CM Hypox (B, D, F) showed that, beside collagen fibrils, the presence of blood vessels full of erythrocytes was evident within the scaffold (circle in A, B). A magnification of the blood vessel displayed in the panel A is shown in Figure C, whilst a capillary, full of erythrocytes, in a longitudinal section, is represented in figure D. Macrophages (arrowheads in E, F) and platelets (asterisk in F) were also present.

### CMs from DPSCs generated in hypoxia have a secretome enriched in pro-angiogenic and monocyte-recruiting/ macrophage-differentiating factors

**Fig. 4:**
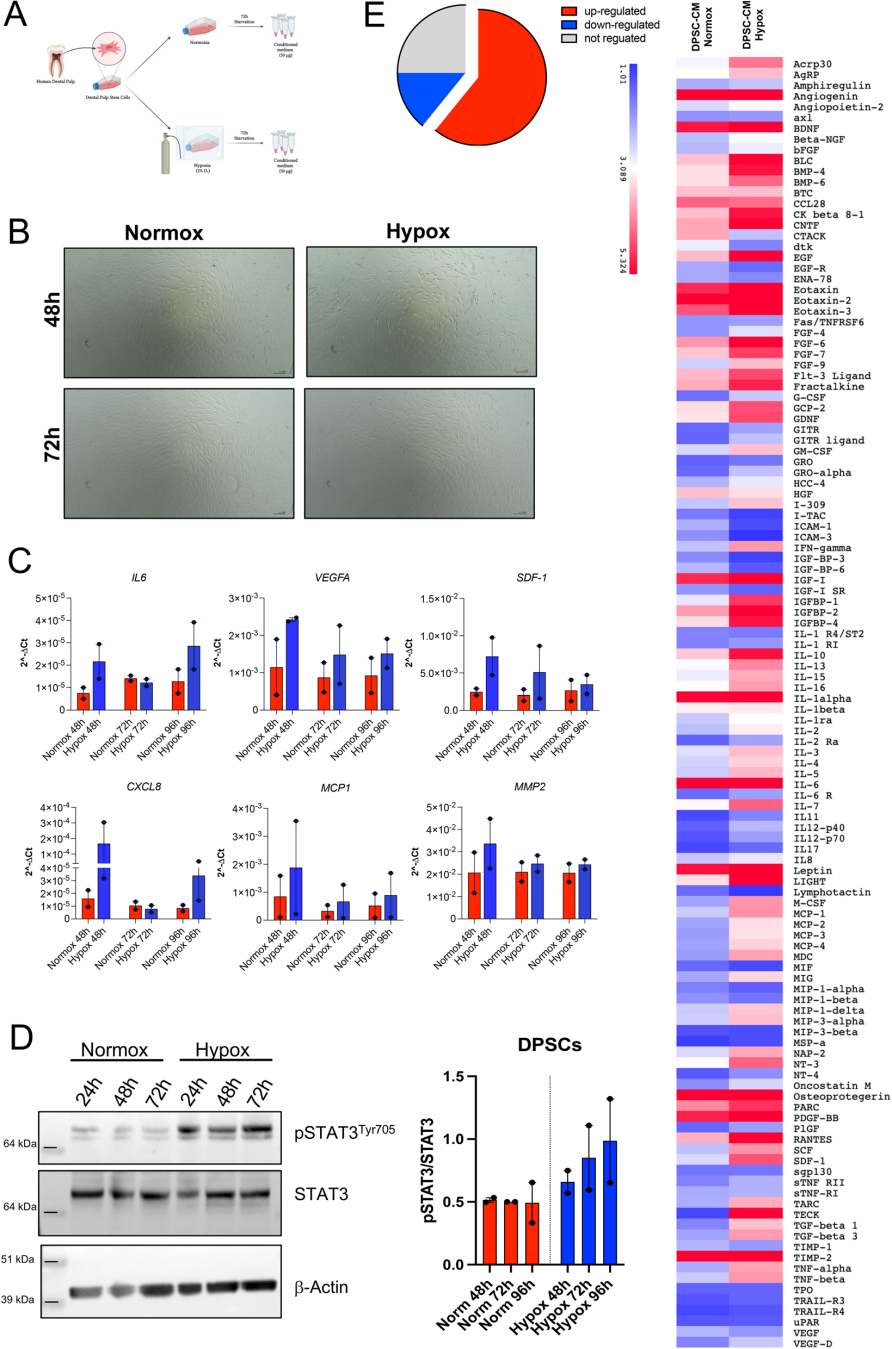
Secretome analysis of DPSC-CM-produced in normoxya and hypoxia. We evaluated the secretome of DPSC-CM Normox and DPSC-CM Hypox by antibody-membrane arrays. **A** Schematic representation for the generation of the DPSC-CMs in normoxia and hypoxia; **B** Representative imagens showing the morphology of DPSCs maintained in normoxia nd hypoxia a 48 and 72 h; **C** qPCR showing the expression levels of hypoxia-inducible target genes (*IL-6, VEGF-A,SDF1, CXCL8, MCP1, MMP2*), on DPSCs cultured in normoxia or hypoxia, for 48–72–96 h; **D** western blot showing the activation of STAT3 pathway, as readout on an hypoxia-mediated signalling, in DPSCs cultured in normoxia or hypoxia, for 48–72–96 h; **E** analysis of the frequency of up, down, and not-regulated factors within the overall arrays and overall heatmap showing the modulation of factors present in C6 and C7 membrane-arrays

Based on the results obtained by the in vivo experiments, we characterized the soluble factors present in the DPSC-CM Normox and DPSC-CM Hypox (Fig. 4A), using commercially available secretome membrane-arrays. We observed that the morphology of DPSCs, maintained both in normoxic and hypoxic conditions, shows no alteration or signs of cell suffering (Fig. 4B) and we confirmed that the hypoxic condition was maintained during all the 72 h of cell culture, as determined by the increased expression of hypoxia-induced genes (*IL-6, VEGF-A, SDF1, CXCL8, MCP1, MMP2*) (Fig. 4C) and increase activation of STAT3 pathway, as a molecular signaling downstream to hypoxia (Fig. 4D).

By secretome analysis, we found that 60,84% of factors were up-regulated in DPSC-CM Hypox compared to DPSC-CM Normox, while 14,16% of factors were down-regulated and 25% of factors shared similar secretion levels (Fig. 4B). Among the most up-regulated soluble factors, we found molecules related to angiogenesis (SDF1, IL-8, FGF4, FGF6, FGF9, VEGFD), monocyte recruitment (MCP1, MCP2, MCP3, MCP4, RANTES, GRO-α, I-309) and monocyte-to-macrophage differentiation (M-CSF, G-CSF, SCF).

### CMs from DPSCs functionally support angiogenesis in vitro

**Fig. 5:**
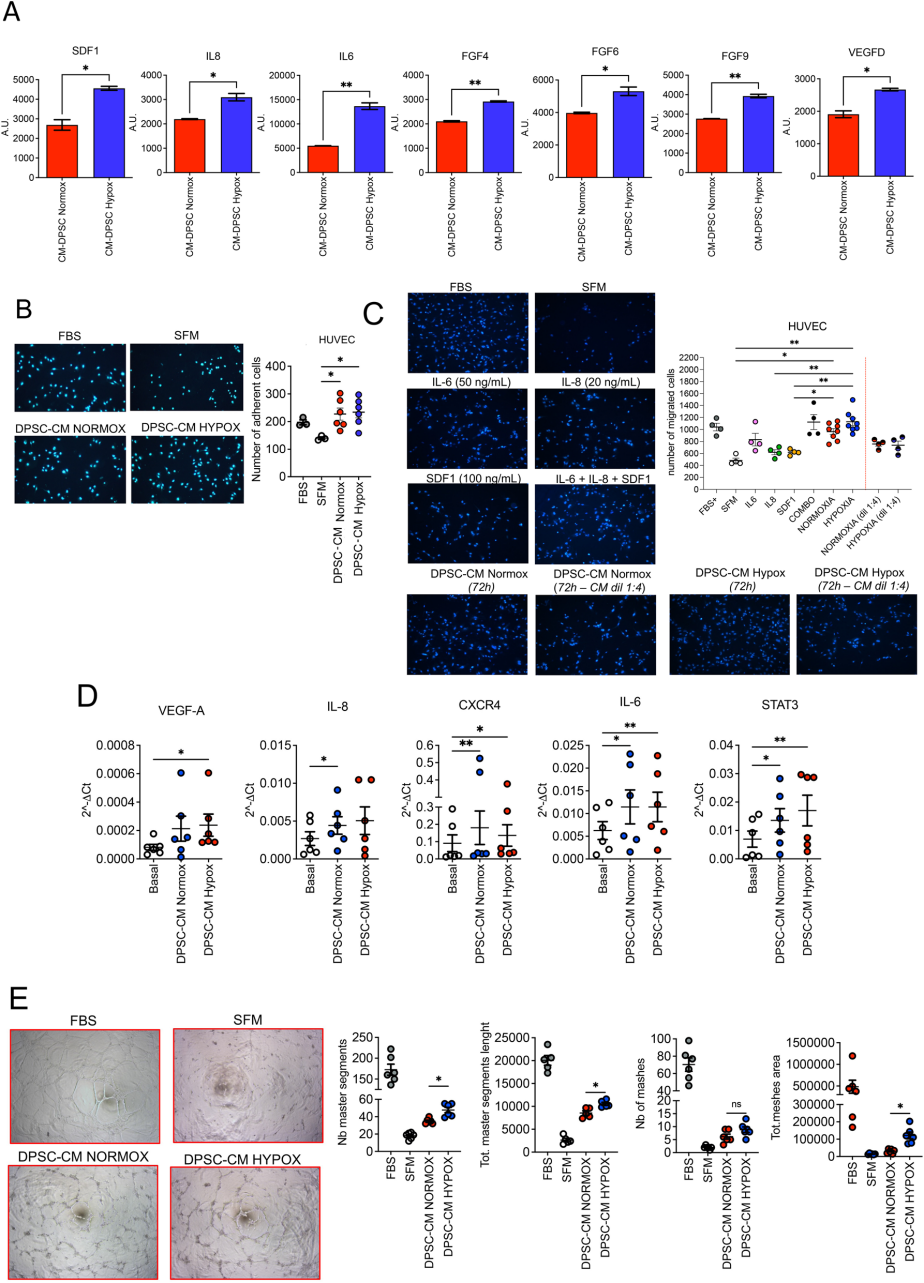
In vitro pro-angiogenic effects of DPSC-CMs produced in normoxia and hypoxia, compared to selected soluble factors*.* The pro-angiogenic effects of DPSC-CM Normox and DPSC-CM Hypox were tested in vitro on Human Umbilical-Vein Endothelial cells (HUVECs). **A** Graphs showing the most up-regulated pro-angiogenic factors in DPSC-CM Normox and DPSC-CM Hypox, as revealed by secretome analysis; **B** detection of HUVEC cell adhesion on fibronectin, upon stimulation with DPSC-CM Normox and DPSC-CM Hypox (magnification 10×); **C** detection of HUVEC cell migration on fibronectin towards DPSC-CM Normox and DPSC-CM Hypox, at 50 μg/mL of total protein or as 1:4 dilution, or IL-6 (50 ng/mL), IL-8 (20 ng/mL), SDF1 (100 ng/mL), or their combination (magnification 10×); **D** real-time PCR for pro-angiogenic factors VEGF-A, IL-8, CXCR4, IL-6, STAT3 expression of HUVECs stimulated with DPSC-CM Normox and DPSC-CM Hypox; **E** tube formation assay on HUVECs stimulated with DPSC-CM Normox and DPSC-CM Hypox (magnification 10×). Results are shown as mean ± SEM, t-student test or One-Way ANOVA, *p ≤ 0.05; **p ≤ 0.01. Basal: basal endothelial cell medium; FBS: foetal bovine serum (10%) supplemented medium; SFM: serum-free medium. Experiments were performed using CMs from 3 donors

Secretome analysis showed increased amounts of the SDF-1, IL-6, IL-8, FGF protein family members (FGF4-6-9) and VEGF-D (Fig. 5A) in DPSC-CM Hypox, compared to DPSC-CM Normox. Therefore, we tested the capability of both CMs to improve angiogenesis on HUVECs. We found that DPSC-CM Normox and DPSC-CM Hypox exhibited similar capabilities to induce HUVEC adhesion on fibronectin (Fig. 5B) and migration (Fig. 5C). When compared with IL-6 or IL-8 or SDF-1, alone, as the major soluble factors enriched in DPSC-CM Hypox (compared to DPSC-CM Normox), the effects of DPSC-CM Hypox was grater, in term of HUVEC migration activities that, in turn, was only comparable with that of the IL-6 + IL-8 + SDF-1 combination (Fig. 5C).

Moreover, when exposed to DPSC-CM Hypox or DPSC-CM Normox, HUVECs were able to increase the expression of different pro-angiogenic factors, namely *VEGF-A*, *IL-8*, *CXCR4*, *IL-6*, *STAT-3* (Fig. 5D). Based on these results, we tested the capability of both CMs to functionally support in vitro angiogenesis by the tube formation assay on Ultimatrix. We found that DPSC-CMs Hypox have increased capability to induce HUVECs to form capillary-like structures compared to DPSC-CMs Normox (Fig. 5E).

### CMs from DPSCs support monocyte recruitment and polarization of M2-like macrophages in vitro

**Fig. 6:**
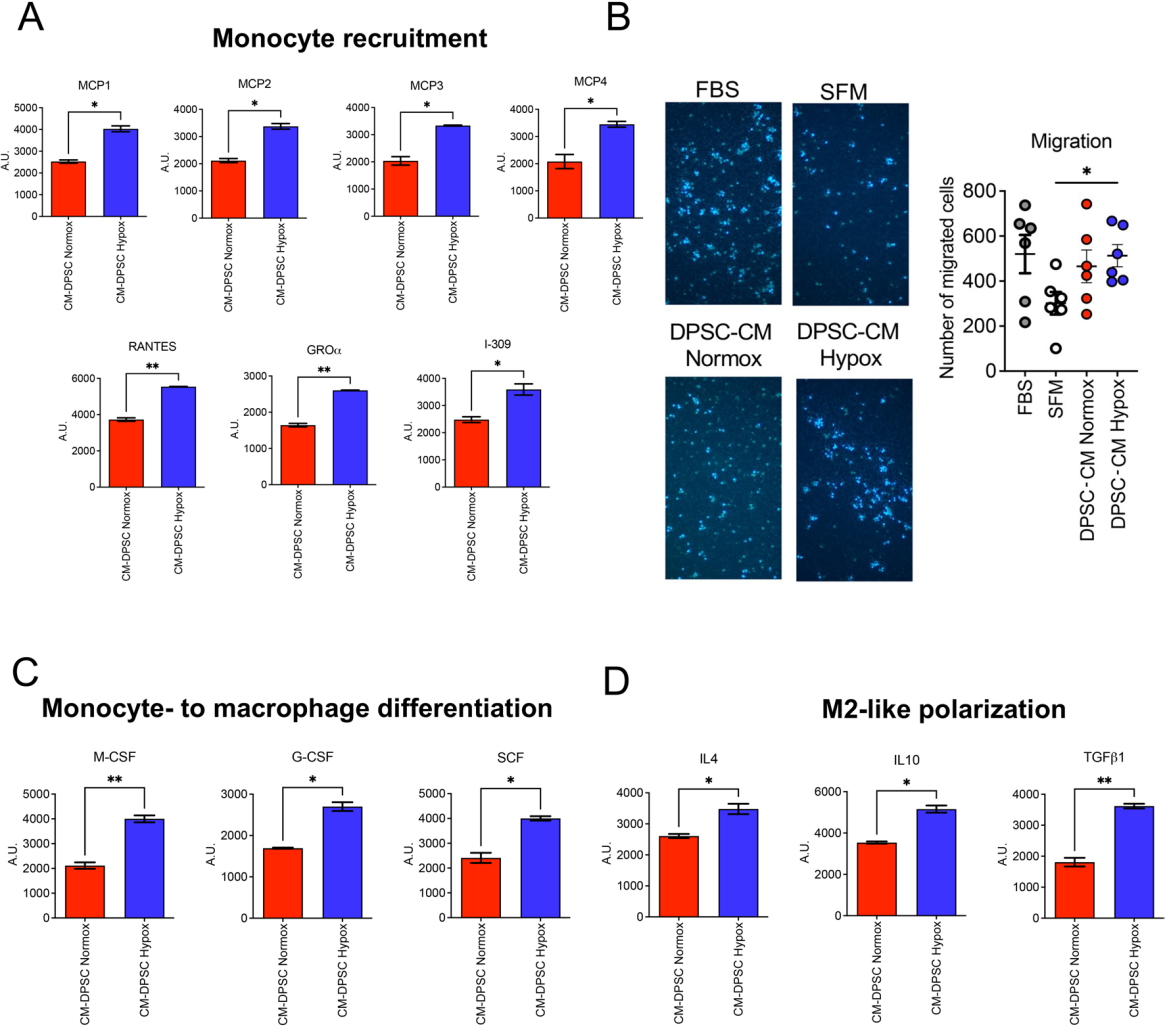
Effects of DPSC-CMs produced in normoxia and hypoxia on monocytes. The effects of DPSC-CM Normox and DPSC-CM Hypox were tested on human CD14^+^ monocytes. Secretome analysis showing the most up-regulated soluble factors in DPSC-CM Normox and DPSC-CM Hypox related to (**A**) monocyte recruitment (MCP1-4, RANTES; GRO-α, I-309); (**B**) effects of DPSC-CM Normox and DPSC-CM Hypox on CD14^+^ monocyte migration (magnification 10×); secretome analysis showing the most up-regulated soluble factors in DPSC-CM Normox and DPSC-CM Hypox related to (**C**) monocyte to-macrophage differentiation (M-CSF, G-CSF, SCF) and (**D**) M2-like polarization (IL-4, IL-10, TGFβ1). Results are shown as mean ± SEM, t-Student test, *p ≤ 0.05; **p ≤ 0.01. FBS: foetal bovine serum (10%) supplemented medium; SFM: serum-free medium

Apart from the increased production of pro-angiogenic factors, secretome analysis also revealed that soluble factors present in DPSC-CM Hypox, such as MCP protein family members (MCP-1–2-3–4), RANTES, GRO-α, and I-309, are involved in monocyte recruitment, (Fig. 6A). We also observed that both DPSC-CM Normox and DPSC-CM Hypox were able to increase the migration activities of human CD14^+^ monocytes (Fig. 6B). Finally, the secretome analysis also revealed that DPSC-CM Hypox are enriched in soluble factors (M-CSF, G-CSF, SCF) involved in monocyte differentiation into macrophages, (Fig. 6C), or implicated (IL-4, IL-10, TGFβ1) in M2-like macrophage polarization (Fig. 6D).

## Discussion

Tissue engineering represents a multidisciplinary field of biomedical science and engineering that involves the generation, repair, or replacement of biological tissues and organs, following injuries.

In this context, stem cells, particularly Mesenchymal Stem Cells (MSCs), are a pivotal tool, owing to their unique capacity for pluripotency and differentiation into diverse cell lineages. Their inherent versatility and regenerative potential render them indispensable for the reconstruction of functional tissues and organs [29, 43, 44].

During the healing phase of the regeneration process, the growth of blood vessels is crucial to supply oxygen and nutrients: thus, the capability of MSCs to support angiogenesis represents a crucial issue.

Recently, we demonstrated that soluble factors derived from Adipose stem cells (ASCs), one of the most used adult stem cells in the field of regenerative medicine [45], showed *in-vivo* and *in-vitro* pro-angiogenic activities that were significantly enhanced in hypoxic condition [38]. In line, we found preliminary evidence that also dental pulp stem cells (DPSCs), similarly to ASCs, can release soluble factors to support in vivo angiogenesis, with a possible involvement of immune cells, particularly by monocytes and macrophages [16]. Therefore, given our previous results, here, we dissect the pro-angiogenic potential of DPSCs, also exploring the impact of hypoxia in promoting their effects on endothelial cells and immune landscape. Since a major concern regarding the use of DPSCs, as direct cell source, is related to their potentially uncontrolled interaction with cells of the host, the main goal of this study was to investigate the in vivo and in vitro pro-angiogenic activities of DPSC secreted soluble factors, (here defined as conditioned media CM), obtained maintaining DPSC in normoxia or hypoxia conditions.

In this study, to recapitulate the heterogeneity of DPSCs and evaluate the variability among donor samples, isolation methods, and culture conditions, that can negatively impact on cell behavior, we used DPSCs from three donors. DPSCs were characterized for MSC prototype markers, showing comparable levels of all markers and proliferation rates, during in vitro maintenance, from passage 2 to passage 30. This aspect is particularly relevant, since prolonged in vitro culture can lead to cellular senescence, characterized by reduced proliferation, that represents a major issue for the use of MSC in regenerative medicine. Together with their differentiation properties, the ability of DPSCs to sustain angiogenesis play a crucial role for their potential use in clinical approaches. Indeed, the formation of new vessels is particularly relevant, during tissue regeneration/repair, and the survival of transplanted or engineered tissues. Therefore, since our major aim was to propose a cell-free, only soluble factors-based approach, for vascular regenerative medicine, we focus our attention on soluble factors released by DPSCs.

Ultimatrix sponges, associated with DPSC-CMs, obtained in hypoxic and normoxic conditions, were then subcutaneously injected into the flank of nude mice. Microscopical inspection of excised sponges showed higher hemoglobin content, vessel count and infiltration of CD31^+^ endothelial cells, in mice injected with DPSC-CM Hypox compared to DPSC-CM Normox, suggesting that, as for ASC-CM, hypoxia was able to promote the release of soluble factors involved in the formation of new vessels. These results were confirmed by ultrastructural analysis, conducted through SEM microscopy, which highlighted that, beside newly formed collagen fibrils, the presence of blood vessels, full of erythrocytes was evident within the scaffold.

We therefore moved to in vitro studies, to better characterize the pro-angiogenic activities of DPSC, via secreted factors, depending on their maintenance in normoxic or hypoxic conditions. DPSCs cultured in hypoxic condition stably maintained the expression of hypoxia-induced genes (*IL-6, VEGF-A, SDF1, CXCL8, MCP1, MMP2*) [46], together with the activation of STAT3 signaling [47], during all the culture conditions (48, 72, 96 h) compared to DPSCs cultured in normoxic conditions.

Accordingly, secretome analysis revealed that hypoxia increased the release of SDF1, IL-8, IL-6, FGFs and VEGF-D, this latter is a soluble factor that has been proposed as a stronger angiogenic driver than VEGF-A [48]. Surprisingly, even if hypoxia was able to increase the pro-angiogenic effects of DPSC-CM in term of tubulogenesis*, *in vitro, no significant differences were observed in pro-migration activities compared to normoxia; these results suggest a major impact of the released factors in hypoxic condition in regulating the assembly of endothelial cells into tube-like structures, rather than an effect on pathways involved in cell motility. In line, HUVECs exposed to DPSC-CM Normox and DPSC-CM Hypox, showed similar increased expression of pro-angiogenic factors, such as VEGF-A, IL-8, CXCR4, IL-6 and STAT-3, compared with basal conditions. Indeed, no differences were observed between hypoxia and normoxia.

To evaluate the contribution of the main pro-angiogenic factors enriched in DPSC-CM, IL-6, IL-8 and SDF1 were tested, showing that only their combination was able to induce an efficient recruitment of HUVEC cells, comparable to DPSC-CM in normoxic and hypoxic conditions, highlighting the relevance of the synergistic action of all soluble factors that cannot be ascribed to a single agent.

The comparable effects obtained in hypoxic and normoxic conditions represent a crucial point that may represents a significant advantage in regenerative medicine, highlighting the flexibility of the proposed application, increasing the range of applications, and increasing time and resource savings (preparation and implementation of DPSC-based therapies). Indeed, while in some application, such ischemic tissue conditions, cells might be under hypoxic conditions, in other cases it may be necessary to induce angiogenesis, even in normally oxygenated tissues, thereby sustaining tissue regeneration. It is widely accepted that, during the repairing phase, some immune cells can acquire pro-angiogenic functions, based on their cell plasticity [2, 5, 6, 9, 10]. We recently demonstrated that in in vivo experiments, DPSCs, used as whole cells or employing their CMs, are able to increase the infiltration of M2-like macrophages, when associated with scaffolds [16]*.* Together with angiogenesis induction, we also observed the recruitment of macrophages within the Ultimatrix sponges, with comparable frequencies of CD45^+^ total leukocytes, F4/80^+^ total macrophages, CD80^+^ M1-macrophages and CD206^+^ M2-macrophages, between DPSC-CMs Hypox and DPSC-CM Normox. Based on the in vivo results, we then proceeded to in vitro experiments, to test the interactions of DPSC-CMs Normox and DPSC-CM Hypox with human CD14^+^ monocytes.

Focusing on soluble factors able to influence monocytes and macrophage phenotype and functions, we found that DPSC-CM Hypox can release higher levels of molecules involved in monocyte recruitment (MCP-1-2-3-4), than DPSC-CMs Normox. Moreover, increased levels of RANTES [49–51], GRO-α [52], I-309 [53] were also found in DPSC-CM Hypox. By contrast, no functional differences were observed when human CD14^+^ monocytes were tested in migration assay, suggesting that other factors contained in CM can influence the migration of immune cells, thereby masking the differences between hypoxia and normoxia. Indeed, the concomitant increase of M-CSF, G-CSF, SCF, involved in monocyte-to-macrophage differentiation, and also soluble factors, such as IL-4, IL-10 and TGFβ1, involved in M2-like polarization [54–56] can suggest that hypoxic conditions may mainly influence macrophage behavior and response to stimuli, rather than recruitment.

Compared to our previous study of the pro-angiogenic activities, via soluble factors, by ASCs, the current study, will impact and gain since: (i) allowed the identification of another possible cellular source of pro-angiogenic factor that can be obtained from a waste material; (ii) increased the available approaches for vascular regenerative medicine, based of cell-free protocols; (iii) allowed proposing the use a human cell source that can be more easily obtained using a minimally invasive procedure, compared to that required to obtain ASCs.

## Conclusions

Overall, our results obtained by in vivo and in vitro experiments, clearly suggest the feasibility of employing soluble factors, derived from DPSCs, as a cell-free device to be used in regenerative medicine for the restoration of a damaged vascular system, a crucial biological event in the healing and repair processes.

## Supplementary Information

**Additional file 1: Table S1**: Sequences of primers used for qPCR analysis. List of the primers and related gene sequences, used in the manuscript.

**Additional file 2: Figure S1**: histological and ultrastructure of Ultimatrix sponges alone or in presence of a cocktail of pro-angiogenic factors. (A) in the sponge alone the blood vessels are absent, but few fibroblasts (arrowheads) have colonized the scaffold; (B) in the sponge combined with VTH (positive control), mature blood vessels (arrows) are noticed; (C) picture shows that in the Ultimatrix alone, the original ultrastructure was maintained; (D) picture of Ultimatrix associated with VTH (positive control) shows newly formed collagen fibrils synthetized by fibroblasts (arrows) that have colonized the scaffold. Scale bar for optical microscopy is 20 μm; scale bars for TEM are indicated in the pictures.

**Additional file 3: Figure S2**: Whole Array for secretome analysis. (A) whole membrane for C6 and C7 arrays; (B) Table showing the correspondence for spot/target for C6 and C7 arrays.

## Data Availability

All data generated or analyzed during this study are available, by the corresponding author on reasonable request.

## Abbreviations

ALPL: Alkaline Phosphatase
ASC: Adipose-derived stem cells
CM: Conditioned media
CXCR4: C-X-C chemokine receptor type 4
DPSC: Dental pulp stem cell
DSPP: Dentin Sialophosphoprotein
ECM: Extracellular matrix
EpCAM: Epithelial cell adhesion molecule
FGF: Fibroblast growth factor
G-CSF: Granulocyte colony-stimulating factor
GF: Growth factor
GRO: Growth regulated protein
IL: Interleukin
MCP: Monocyte Chemoattractant Protein
M-CSF: Macrophage colony-stimulating factor
MSC: Mesenchymal stem cell
RANTES: Regulated on activation, normal T cell expressed and secreted
SCF: Stem cell factor
SDF-1: Stroma cell derived factor
STAT-3: Signal transducer and activator of transcription 3
TGFβ: Tumor growth factor beta
VEGF: Vascular endothelial growth factor
