# Supplementary material for: Dental pulp mesenchymal stem cell (DPSCs)-derived soluble factors, produced under hypoxic conditions, support angiogenesis via endothelial cell activation and generation of M2-like macrophages
Source: J Biomed Sci. 2024 Nov 4;31:99. doi: 10.1186/s12929-024-01087-6 (PMC11533415; doi:10.1186/s12929-024-01087-6)
Supplement: Supplementary file 1 — Additional file 1: Table S1: Sequences of primers used for qPCR analysis. List of the primers and related gene sequences, used in the manuscript. [file 12929_2024_1087_MOESM1_ESM.docx]

**SUPPLEMENTARY METHODS**

**Matrices**

The *in vivo* angiogenesis matrix sponge assay was performed using the Cultrex Ultimatrix (Biotechne, Minneapolis, MN, USA) at 10 mg/mL of concentration (1).

**CD14^+^monocyte isolation and maintenance**

CD14^+^ monocytes were isolated from blood samples of healthy-donor volunteers recruited within the protocol n° 463.2021, approved by the IRCCS MultiMedica internal Ethical Committee, according to the Helsinki Declaration of 1975 as revised in 2013.

DPSCs were isolated as reported in Barone et al. (2) ; briefly the dental pulp was digested through 3 mg/mL collagenase type II (Sigma Aldrich, Milano, Italy) at 37 °C for 1 h, under agitation. The obtained fraction was filtered with a 70 µm cell strainer and then centrifuged at 960×g for 10 min (Neya 8—Remi Elektrotechnik Ltd., Mumbai, India). The resulting pellet was then resuspended in culture medium, DMEM:DMEM F12 1:1 (Sigma Aldrich, Milano, Italy), supplemented with 2 mM L-Gln, 1% penicillin–streptomycin, 0.1% gentamicin, and 10% FBS, and cultured in T25 flasks at 37 °C, 5% CO_2_. After 24 h, unattached cells were removed. All experiments were conducted at passage five, which is considered as an early passage, to obtain a huge number of DPSCs (3).

Human CD14^+^ monocytes were obtained from peripheral blood mononuclear cells (PBMCs) from healthy individuals, by density gradient stratification on ficoll hystopaque (Sigma Aldrich). PBMCs were subjected to positive magnetic selection for total CD14^+^monocytes, using CD14 micro-beads (Miltenyi Biotech), following the manufacturer’s instructions.

**Cell line culture and maintenance**

Human Umbilical-Vein Endothelial Cells (HUVECs, ATCC) were cultured in endothelial cell basal medium (EBM, Lonza) supplemented with endothelial cell growth medium SingleQuots and Growth Factors (Lonza), 2 mM l-glutamine (Euroclone), 100 U/mL penicillin, and 100 μg/mL streptomycin (Euroclone). Cells were maintained at 37 °C with 5% CO_2_. HUVECs within passages 3-4 were utilized in the experiments. Regular screening was performed to confirm the absence of mycoplasma contamination in all cell types.

**Characterization of DPSC-conditioned media**

The DPSC-CMs were characterized, in term of soluble factor content, using the Human Cytokine Array C7 (RayBiotec) (Peachtree Corners, GA, USA), starting from 50 μg of total protein of DPSC-CMs from normoxic or hypoxic conditions and following the manufacturer’s indication and as in (4-6). Signal intensity, as chemiluminescent dots, was detected using the Alliance Q9 instrument (UVITEC). Arrays were computed scanned using the Alliance Q9 instrument (UVITEC), and optical density, for each dot/spot, was determined using the ImageJ software and Dot plot Analyzer plugin.

***In vivo* Ultimatrix sponge assay**

Male athymic BALB/c nude Crl:CD1-Foxn1nu086 mice (Charles River mice, seven weeks), were employed in the study. Each mouse (9 animals per experimental group) received a subcutaneous injection of 600 μl of liquid matrigel, combined with 50 μg of total protein of either DPSC-CMs generated in normoxia or hypoxia, into the right and left flanks. Mice were housed under standard conditions with a 12-hour light/dark cycle and provided ad libitum access to food and water. The experiments were conducted in compliance with the guidelines established by the Italian and European Community (D.L. 2711/92 No.116; 86/609/EEC Directive), adhering to the principles of the 3 Rs (Replacement, Reduction, and Refinement) and carried out within an approved protocol by the institutional ethics committee. After six days post-injections, Ultimatrix sponges were excised from the mice and utilized for measuring hemoglobin levels, conducting histological analysis, and performing Fluorescence Activated Cell Sorting (FACS) analysis.

**Real-Time PCR**

DPSCs from the three donors were collected at passages 2, 5, 10, 20, and 30 and characterized by qPCR. Total RNA extraction was performed by Trizol protocol and subsequently quantified by the QuantiFluor® RNA System (Promega, Milano, Italy); its integrity was assessed by 1% agarose gel electrophoresis. The iScript™ cDNA Synthesis Kit (BioRad, Milano, Italy) allowed the RNA transcription, and the obtained cDNA was stored at −20 °C until use. qPCR was performed using iTaq Universal SYBR® Green Supermix (BioRad, Milano, Italy) and specific primers for stem markers CD44, CD90, and CD105, leukocyte marker CD45, differentiation markers Alkaline Phosphatase (ALPL) and Dentin Sialophosphoprotein (DSPP), and cell senescence markers p16 and p21. The Beacon Designer Program (BioRad, Milano, Italy) allowed the design of primers used for this experimental plan (sequences are shown in Supplementary Table 1). Each sample was prepared as reported in (7). Briefly, 1 µL (5 ng) of cDNA, 1 µL of forward and reverse primer mix (6 µM), 7.5 µL of SYBR Green Supermix (2×), and water to a final volume of 15 µL were mixed and placed in the CFX 96 Thermocycler (BioRad, Milano, Italy). Values were normalized with two reference genes, glyceraldehyde-3-phosphate dehydrogenase (GAPDH) and β-actin, according to the method in (8) and quantified by using the ΔCt method. Each experiment was repeated three times.

HUVECs, following 6 hours of stimulation with 50 μg CMs, were collected in trizol reagent, and stored at -80 °C until further use. Total RNA was extracted using the small RNA miRNeasy Mini Kit (Thermo Fisher) and its concentration was determined using a Nanodrop Spectrophotometer (Thermo Fisher). Reverse transcription of 500 ng of total RNA was performed using the SuperScript VILO cDNA synthesis kit (Thermo Fisher Scientific, USA). Real-time PCR analysis was conducted using the SYBR Green Master Mix (Thermo Fisher) on the QuantStudio 6 Flex Real-Time PCR System Software (Thermo Fisher). The primer sequences can be found in Supplemental Table 1.

**Western blot**

Cell lysates were prepared using RIPA buffer, supplemented with protease and phosphatase inhibitor cocktails (Roche Diagnostics GmbH). Proteins (30 µg) were separated on a NuPAGE Novex 4%–12% Bis-Tris Gel (Life Technologies) and then transferred to an Amersham Hybond Nitrocellulose membrane (GE Healthcare Bio-Sciences, Pittsburgh, PA, USA). The membranes were incubated overnight at 4°C with primary anti-human STAT3 or anti-human pSTAT3^Tyr705^ antibodies (Cell Singaling, 1:1,000 dilution). Membranes were then washed three times with TBS containing 0.05% Tween 20 (TBS-T, pH 7.4) and incubated for 1 hour at room temperature with a peroxidase-linked anti-rabbit IgG secondary antibody (GE Healthcare Bio-Sciences, Pittsburgh, PA, USA, 1:2,000 dilution). Protein bands were visualized using the Alliance Q9 Atom System (Uvitec, Cambridge, UK). STAT3 and pSTAT3 protein levels were normalized on β-actin (Cell Signaling Technology, 1:1,000 dilution) and the pSTAT3/STAT3 ratio was showed in quantification bat graph. Western blot results were analyzed with Q9 Alliance software (Uvitec, Cambridge, UK) to measure the band optical density (OD).

**Table 1: Sequences of primers used for qPCR analysis.**

| **Primers target** | **Forward** | **Reverse** |
| --- | --- | --- |
| *Hs_CD44* | GCAGTCAACAGTCGAAGAAG | GTCCTCCACAGCTCCATT |
| *Hs_CD90* | CTCTACTTATCCGCCTTCACT | CGTTCTGGGAGGAGATGG |
| *Hs_CD105* | AGGCGGTGGTCAATATCC | GTGTGGGCTGAGGTAGAG |
| *Hs_CD45* | ACAGGAACCTATATCGGAATTGA | TTGAACCATCAGGCATCTCT |
| *Hs_ALPL* | TGAGTGACACAGACAAGAAG | CTGGTAGTTGTTGTGAGCATA |
| *Hs_DSPP* | GCAGTGACAGTGATAGTAGTG | TTGCTGCTGTCTGACTTG |
| *Hs_p16* | CATAGATGCCGCGGA AGGT | CTA AGTTTCCCGAGGTTTCTCAGA |
| *Hs_p21* | GTT CTA CCT CAG GCA GCT CA | AAT GAA CTG GGG AGG GAT GG |
| *hCXCR4* | ATCAGTCTGGACCGCTACCT | CCACCTTTTCAGCCAACAGC |
| *hIL-6* | GAGGAACAAGCCAGAGCTG | GGTCAGGGGTGGTTATTGC |
| *hIL-8* | CCTGATTTCTGCCAGCTCTGTG | GTGGTCCACTCTCAATCACTCTC |
| *hVEGFA* | TTGCCTTGCTGCTCTACCTCCA | GATGGCAGTAGCTGCGCTGATA |
| *hSTAT-3* | CTTTGAGACCGAGGTGTATCACC | GGTCAGCATGTTGTACCACAGG |
| *hSDF-1* | AACGCCAAGGTCGTGGTC | GAATCGGCATGGGCATCTGT |
| *MCP-1* | AGAGGCTGAGACTAACCCAGA | TTTCATGCTGGAGGCGAGAG |
| *h18S* | GCAGAATCCACGCCAGTACAAG | GCTTGTTGTCCAGACCATTGGC |
| *Hs_ACTβ* | GAAGATCAAGATCATTGCTCCTC | GACTCGTCATACTCCTGCTT |
| *Hs_GAPDH* | ATCATCAGCAATGCCTCCT | GAGTCCTTCCACGATACCAA |

**References.**

1. Benedetto N, Calabrone L, Gutmanska K, Macri N, Cerrito MG, Ricotta R, et al. An Olive Oil Mill Wastewater Extract Improves Chemotherapeutic Activity Against Breast Cancer Cells While Protecting From Cardiotoxicity. Front Cardiovasc Med. 2022;9:867867.

2. Barone L, Gallazzi M, Rossi F, Papait R, Raspanti M, Zecca PA, et al. Human Dental Pulp Mesenchymal Stem Cell-Derived Soluble Factors Combined with a Nanostructured Scaffold Support the Generation of a Vascular Network In Vivo. Nanomaterials (Basel). 2023;13(17).

3. Ratushnyy A, Ezdakova M, Buravkova L. Secretome of Senescent Adipose-Derived Mesenchymal Stem Cells Negatively Regulates Angiogenesis. Int J Mol Sci. 2020;21(5).

4. Bruno A, Bassani B, D'Urso DG, Pitaku I, Cassinotti E, Pelosi G, et al. Angiogenin and the MMP9-TIMP2 axis are up-regulated in proangiogenic, decidual NK-like cells from patients with colorectal cancer. FASEB J. 2018;32(10):5365-77.

5. Gallazzi M, Baci D, Mortara L, Bosi A, Buono G, Naselli A, et al. Prostate Cancer Peripheral Blood NK Cells Show Enhanced CD9, CD49a, CXCR4, CXCL8, MMP-9 Production and Secrete Monocyte-Recruiting and Polarizing Factors. Front Immunol. 2020;11:586126.

6. Barone L, Palano MT, Gallazzi M, Cucchiara M, Rossi F, Borgese M, et al. Adipose mesenchymal stem cell-derived soluble factors, produced under hypoxic condition, efficiently support in vivo angiogenesis. Cell Death Discov. 2023;9(1):174.

7. Rossi F, Bernardini G, Bonfanti P, Colombo A, Prati M, Gornati R. Effects of TCDD on spermatogenesis related factor-2 (SRF-2): gene expression in Xenopus. Toxicol Lett. 2009;191(2-3):189-94.

8. Palombella S, Pirrone C, Cherubino M, Valdatta L, Bernardini G, Gornati R. Identification of reference genes for qPCR analysis during hASC long culture maintenance. PLoS One. 2017;12(2):e0170918.
