# Supplementary figures and images for: Dental pulp mesenchymal stem cell (DPSCs)-derived soluble factors, produced under hypoxic conditions, support angiogenesis via endothelial cell activation and generation of M2-like macrophages
Source: J Biomed Sci. 2024 Nov 4;31:99. doi: 10.1186/s12929-024-01087-6 (PMC11533415; doi:10.1186/s12929-024-01087-6)

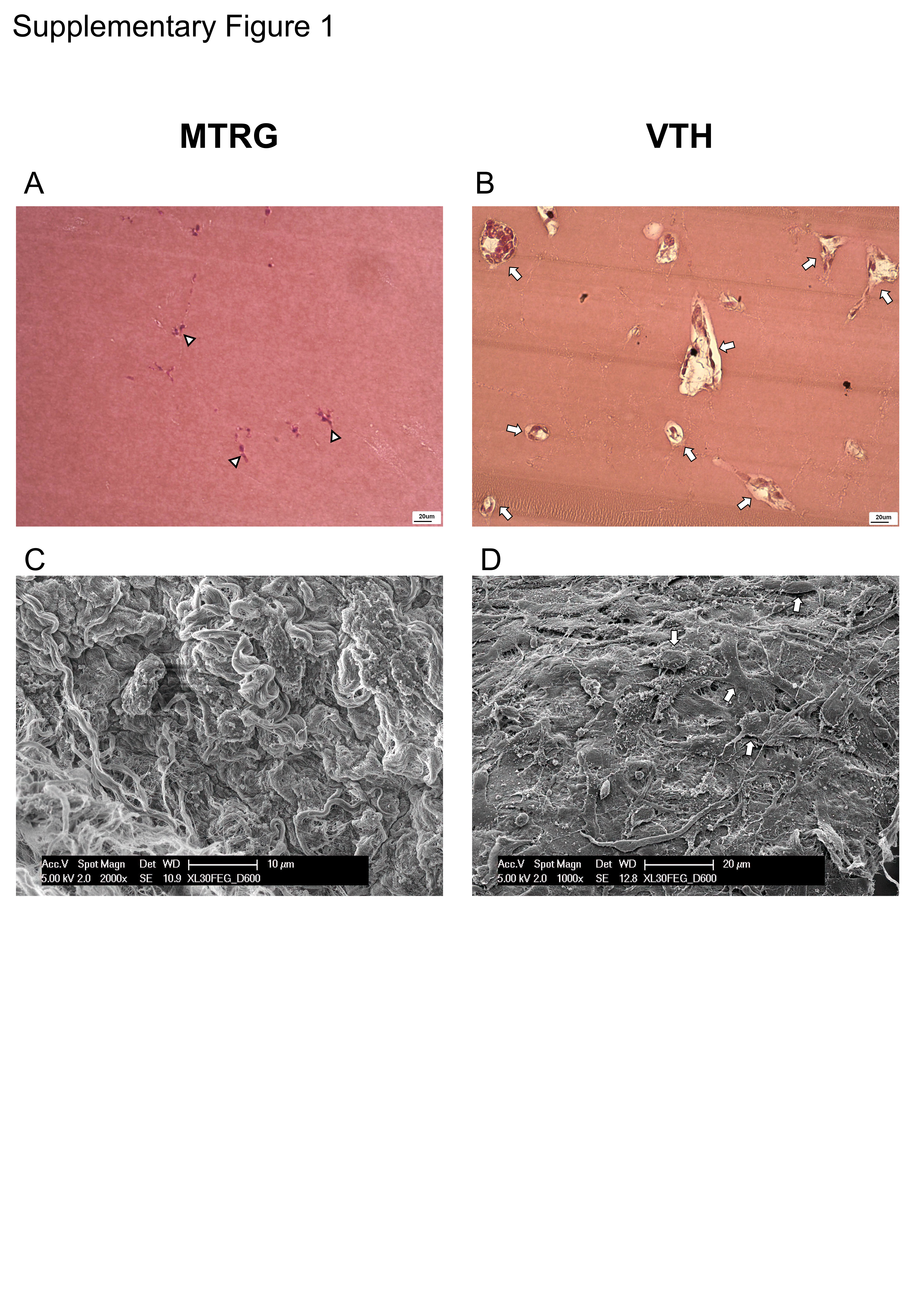

Supplement: Supplementary file 2 — Additional file 2: Figure S1: histological and ultrastructure of Ultimatrix sponges alone or in presence of a cocktail of pro-angiogenic factors. (A) in the sponge alone the blood vessels are absent, but few fibroblasts (arrowheads) have colonized the scaffold; (B) in the sponge combined with VTH (positive control), mature blood vessels (arrows) are noticed; (C) picture shows that in the Ultimatrix alone, the original ultrastructure was maintained; (D) picture of Ultimatrix associated with VTH (positive control) shows newly formed collagen fibrils synthetized by fibroblasts (arrows) that have colonized the scaffold. Scale bar for optical microscopy is 20 μm; scale bars for TEM are indicated in the pictures. [file 12929_2024_1087_MOESM2_ESM.jpg]

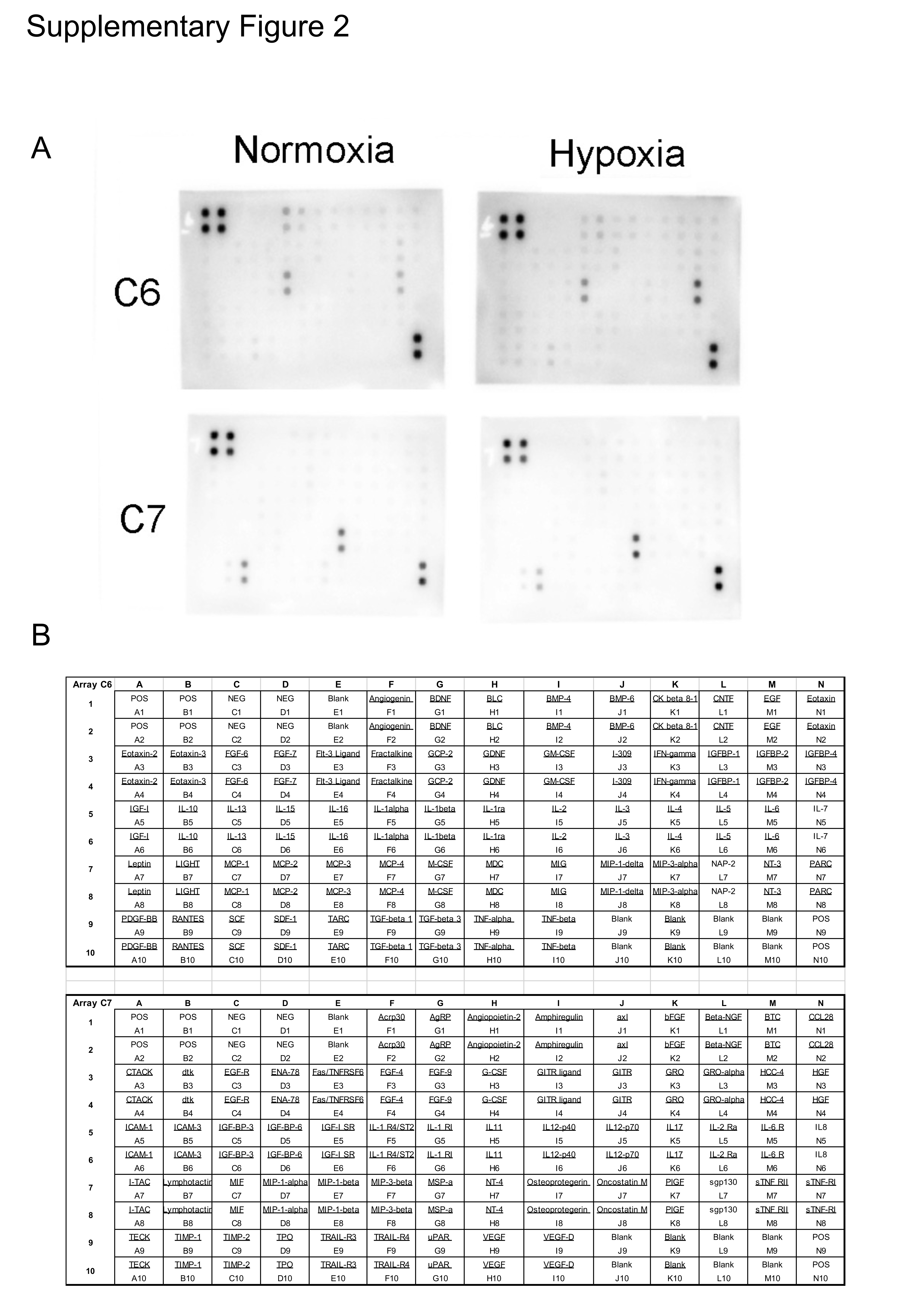

Supplement: Supplementary file 3 — Additional file 3: Figure S2: Whole Array for secretome analysis. (A) whole membrane for C6 and C7 arrays; (B) Table showing the correspondence for spot/target for C6 and C7 arrays. [file 12929_2024_1087_MOESM3_ESM.jpg]
